# Pretreatment of trans-ferulic acid improved neurotoxicity in a thioacetamide-induced hepatic encephalopathy rat model; participation of the MAPK/NF-κb signaling pathway

**DOI:** 10.3389/fphar.2026.1750709

**Published:** 2026-05-21

**Authors:** Adil Furkan Kilic, Bahadir Suleyman, Renad Mammadov, Seval Bulut, Ali Sefa Mendil, Halis Suleyman

**Affiliations:** 1 Department of Internal Medicine, Erzurum Faculty of Medicine, Health Sciences University, Erzurum, Türkiye; 2 Department of Pharmacology, Faculty of Medicine, Erzincan Binali Yildirim University, Erzincan, Türkiye; 3 Department of Pathology, Faculty of Veterinary Medicine, Erciyes University, Kayseri, Türkiye

**Keywords:** hepatic encephalopathy, MAPK/NF-κB, rats, thioacetamide, trans-ferulic acid

## Abstract

**Aim:**

Trans-ferulic acid (TFA), a naturally occurring phenolic compound, has attracted significant attention due to its strong hepatoprotective and neuroprotective properties. Despite extensive research on its overall biological activities, the specific mechanisms by which TFA influences hepatic encephalopathy (HE) remain unclear. This study aims to explore the therapeutic potential of TFA by examining its effects on inflammatory signaling, glial activation, and apoptotic pathways in a thioacetamide (TAA)-induced rat model of HE.

**Methods:**

Rats were separated into four groups: a normal control group, the TAA group (which received TAA at 300 mg/kg/day for 3 days), and groups pre-treated with TFA (at doses of 25/50 mg/kg, administered orally) for 10 days, with TAA administered from the eighth to the 10th day.

**Results:**

TFA pre-treatment effectively reduced TAA-induced biochemical disturbances, as shown by lower serum levels of aspartate aminotransferase (AST), alanine aminotransferase (ALT), alkaline phosphatase (ALP), and ammonia. This biochemical improvement was complemented by a significant reduction in TAA-related hepatic histopathological damage. TFA also alleviated systemic inflammation by suppressing TAA-induced increases in pro-inflammatory mediators, including tumor necrosis factor-alpha (TNF-α) and interleukin-6 (IL-6), while also elevating the anti-inflammatory cytokine IL-10. Consistent with these results, TFA further suppressed the activation of MAPK/NF-κB inflammatory signaling pathways in both liver and brain tissues and decreased glial fibrillary acidic protein (GFAP) expression in the brains of TAA-treated rats, indicating reduced neuroinflammation and astrocyte activation. Additionally, immunochemical analysis showed that TFA pre-treatment effectively prevented the TAA-induced increase in pro-apoptotic markers Caspase-3 and Bax in both liver and brain tissues. This study underscores the strong protective effects of TFA against TAA-induced HE, mainly through inhibition of MAPK/NF-κB signaling, suppression of astrocyte activation, and reduction of apoptosis.

**Conclusion:**

These outcomes imply that TFA could be an effective treatment for HE.

## Graphical Abstract

**Figure:**
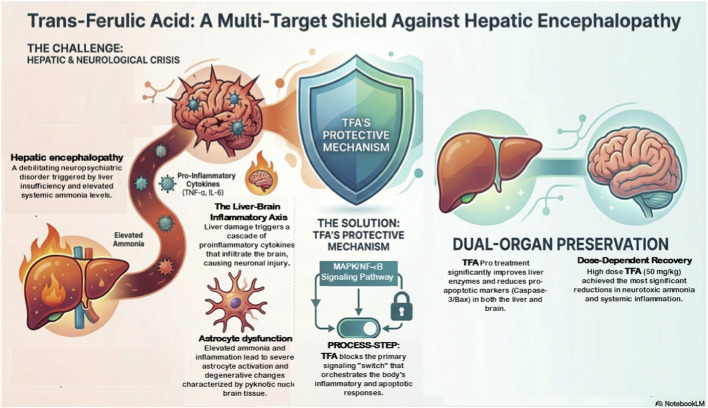

## 1 Introduction

Hepatic encephalopathy (HE) is a neurological disorder caused by liver failure, which greatly reduces patients’ quality of life (Cordoba, 2014). The condition results from complex molecular processes such as ammonia toxicity, inflammation, and oxidative stress, making treatment difficult. Due to the limited success of existing therapies, there is an urgent need for new options that can halt or reverse the disease’s progression (Ali and Datusalia, 2025; Cordoba, 2014; Ntuli and Shawcross, 2024).

The current dominant paradigm in HE pathogenesis implicates hyperammonemia secondary to hepatic insufficiency as a central neurotoxic driver, initiating astrocytic dysfunction. This, in conjunction with ammonia-induced activation of proinflammatory and oxidative stress pathways, culminates in progressive neuronal injury (Bao et al., 2025; Gallego-Durán et al., 2024). Various mechanisms contributing to these cellular disruptions have been studied as potential targets for therapy (Gad et al., 2025). Notably, research has highlighted mitogen-activated protein kinases (MAPKs) and nuclear factor-kappa B (NF-κB) as key signaling molecules involved in modulating ammonia-induced neuroinflammation in variegated pre-clinical and clinical studies (Ali and Datusalia, 2024; Balzano et al., 2020; Khalil et al., 2023). Furthermore, activated microglial cells demonstrated triggering of MAPK/NF-κB signaling, which actuated apoptotic cascades (Shen et al., 2024). The interactive progression of these pathophysiological processes can lead to neuronal dysfunction and structural damage, thereby exacerbating the neurodegenerative nature of HE (Ali and Datusalia, 2024; 2025). Therefore, considering the role of inflammatory signaling in HE, targeting the MAPK/NF-κB pathway could aid in developing more effective therapeutic strategies. This report presents a novel preventive approach utilizing trans-ferulic acid to target inflammatory mechanisms in HE.

Trans-ferulic acid (TFA), a phenolic compound originating from hydroxycinnamic acid, has gained considerable interest in the pharmacology field due to its antioxidant and therapeutic features (Singh and Patil, 2022). TFA is reported to have hepatoprotective effects by suppressing oxidative damage, inflammatory response, and apoptotic cell death in different experimental liver injury models (Mahmoud et al., 2020; Nouri et al., 2023). In the literature, pre-treatment with ferulic acid has been reported to exert a protective effect against methotrexate-induced liver injury, with emphasis on its anti-inflammatory properties (Roghani et al., 2020). Moreover, TFA shows neuroprotective effects such as improving memory (Park et al., 2022), strengthening synaptic plasticity (Mahaman et al., 2023), and alleviating neuroinflammation by suppressing the production of inflammatory molecules (Kinra et al., 2024; Wang et al., 2023). In an experimental study, ferulic acid was shown to exert anti-inflammatory and neuroprotective effects in lipopolysaccharide-induced neuroinflammation, thereby reducing proinflammatory cytokine release and improving sickness behavior (Kinra et al., 2024). Building upon these findings, we hypothesized that TFA may confer protective effects against HE by modulating inflammatory signaling pathways and reducing apoptosis, thereby attenuating neuronal damage secondary to liver dysfunction. To this end, the present study investigated the potential of TFA to ameliorate pathological alterations associated with experimentally induced HE in rats, focusing particularly on its anti-inflammatory and anti-apoptotic effects mediated through the MAPK/NF-κB signaling pathway. To date, the effects of TFA on the pathophysiology of HE have not been explored. This study intends to address this by elucidating, for the first time, the potential protective mechanisms of TFA in an experimental HE model. The findings are expected to contribute valuable preclinical insights into the translational capacity of TFA in the development of novel therapeutic strategies for HE.

## 2 Materials and methods

### 2.1 Chemicals and natural compounds

TAA (CAS Number # 62–55–5), trans-ferulic acid (CAS Number: 537–98–4), and sodium pentobarbital were obtained from Sigma-Aldrich (Darmstadt, Germany).

### 2.2 Experimental animals

Forty male Sprague Dawley rats (age: 2 months; body weight: 160 ± 20 g) were obtained from the Experimental Animals Application and Research Center of Erzincan Binali Yildirim University. Animals were housed in polypropylene cages under controlled environmental circumstances (22 °C ± 1 °C; 12 h light/12 h dark). During the trial, all rats were given as much food and water as they wanted. All experimental procedures were conducted in accordance with the ethical guidelines for the care and use of laboratory animals, which were developed to ensure the highest standards of animal welfare.

### 2.3 Induction of hepatic encephalopathy

The rat model of hepatic encephalopathy (HE) was developed according to previously described methodologies and is consistent with a model of acute liver failure–induced acute hepatic encephalopathy (Type A), in accordance with the ISHEN animal model guidelines (DeMorrow et al., 2021; Ali and Datusalia, 2024). Thioacetamide (TAA) was administered intraperitoneally (i.p) to the experimental animals at a dosage of 300 mg/kg/day over three consecutive days. TAA was freshly prepared in a 0.9% saline solution immediately prior to administration. To mitigate the risk of renal failure, hypoglycaemia, and electrolyte disturbances, 0.5 mL of Lactated Ringer’s solution, supplemented with 10% dextrose, was administered subcutaneously at 12-h intervals following each TAA injection (Okkay, Ferah Okkay, Cicek, Aydin and Ozkaraca, 2022).

### 2.4 Experimental design

The animals were randomly allocated into four groups (n = 10 per group). **Group I** (Control) received the vehicle for TFA (0.5% carboxymethyl cellulose; CMC) orally once daily for 10 consecutive days, along with the vehicle for TAA (normal saline) administered intraperitoneally from day 8 to day 10. **Group II (**TAA) received 0.5% CMC, the vehicle for TFA, once daily for 10 days, with TAA administered i. p at a dose of 300 mg/kg from days 8 to 10 of the experiment. **Group III** (TFA25+ TAA) received TFA suspended in 0.5 CMC at a dose of 25 mg/kg/day, orally once daily for 10 days concurrently with an i. p TAA injection of 300 mg/kg from days 8 to 10. **Group IV** (TFA50+ TAA) received TFA suspended in 0.5 CMC at a dose of 50 mg/kg/day, orally once daily for 10 days concurrently with an i. p TAA injection of 300 mg/kg from days 8 to 10 (Ali and Datusalia, 2025). TFA dosing regimens were established based on evidence from prior investigations (Bhuia et al., 2024; Hassanein et al., 2021). The use of TFA as a pretreatment, as well as the selection of its therapeutic doses, was guided by evidence from previous studies (Mahmoud et al., 2020). TFA has previously been applied as a pretreatment in liver and testicular injuries mediated by inflammatory mechanisms, and it has been demonstrated to confer protective effects on these tissues (Mahmoud et al., 2020; Hassanein et al., 2021). In a previous study, rats treated with ferulic acid alone showed liver function parameters and hepatic inflammatory markers similar to those of healthy controls (Nouri et al., 2023). Animals included in the final analysis were determined based on completion of the experimental protocol and the availability of complete biochemical, histopathological, and immunohistochemical data. Consequently, the study was completed with six animals per group.

Twenty-four hours following the administration of the final dose, 24 hours following the administration of the final dose, the animals were rendered unconscious with sodium pentobarbital (30 mg/kg, i. p.) and subsequently euthanized via exsanguination through intracardiac blood collection into anticoagulant-containing tubes. Blood samples were centrifuged (3,000 rpm, 5 min) to separate the serum, which was then aliquoted and kept at −80 °C for biochemical assessments. Following dissection, liver and brain tissues were harvested and immediately rinsed with ice-cold phosphate-buffered saline (PBS, pH 7.4) to rinse off any blood. For histopathological and immunohistochemical evaluation, a portion of the hepatic lobe and the prefrontal cortex were fixed in 10% neutral-buffered formalin. The remaining tissue samples were homogenized at a ratio of 100 mg tissue to 900 µL ice-cold PBS (pH 7.4) containing 1X protease inhibitor cocktail. For this purpose, 9 µL of 100X protease inhibitor cocktail was added per 900 µL PBS immediately before homogenization. Homogenates were centrifuged at 1800 *g* for 10 min at 4 °C, and clear filtrate was collected and stored at −80 °C for biochemical assays via enzyme-linked immunosorbent assay (ELISA).

### 2.5 Biochemical analyses

Rat serum activities of aspartate aminotransferase (AST), alanine aminotransferase (ALT), and alkaline phosphatase (ALP) (Elabscience, United States; Cat no: E-EL-R0076; ALT; Cat no: E-EL-R1232; Cat no: E-BC-K091-M) were quantified using commercially available assay kits following the manufacturer’s protocols to assess hepatic injury.

To evaluate TAA-associated inflammation, serum levels of tumor necrosis factor-alpha (TNF-α), interleukin-6 (IL-6), and IL-10 were measured using commercial ELISA kits (Elabscience, United States; Cat no: E-EL-H0105, E-EL-H0102, and E-EL-H0108, respectively). ELISA assays were performed according to the manufacturer’s instructions. Cytokine concentrations were calculated based on standard curves generated for each assay.

MAPK (p38) and NF-κB levels in hepatic and brain tissues were quantified using commercially available ELISA kits specific for rat (MAPK (p38) [Cat no: 201–11-1410] and NF-κB [Cat no: 201–11-0288], Sunred Bioscience, Shanghai, China) following the manufacturer’s protocols. Protein concentrations were calculated based on the standard curves prepared in each assay and normalized to total protein content.

Protein content was assessed using the Bradford method by measuring absorbance at 595 nm (Bradford, 1976).

### 2.6 Histopathological examination

For methodological standardization, the right lobes of the liver were selected for histopathological evaluation (Abd Elrazik and Abd El Salam, 2024), while the prefrontal cortex was chosen to assess the cerebral effects of Hepatic encephalopathy. The prefrontal cortex was specifically targeted due to its central role in executive cognitive functions and its susceptibility to hyperammonemia-induced neuroinflammation and neuronal dysfunction associated with this condition (Wang et al., 2025). After the experimental period, liver and brain tissues were fixed in 10% formaldehyde solution for 48 h to preserve cellular architecture and prevent autolysis. Subsequently, the specimens were dehydrated using a graded ethanol series, cleared in xylene, and embedded in paraffin. Then, 4 μm sections were obtained using a microtome and stained with hematoxylin and eosin (H&E) to facilitate histopathological assessment under a light microscope. Histopathological evaluation was performed at ×40 magnification in six randomly selected fields. Liver and prefrontal cortex tissues alterations were assessed using a semi-quantitative scoring system ranging from 0 to 3 based on previously described criteria. Liver tissues were histopathologically evaluated for the presence of hydropic degeneration and mononuclear cell infiltration in the periportal regions (Bilici et al., 2023). A score of 0 indicated normal histological architecture with no pathological alterations. In liver tissue, 1 indicated mild changes with focal lesions affecting less than 25% of hepatic lobules (score 1), 2 indicated moderate changes with lesions involving up to 50% of hepatic lobules (score 2), and 3 indicated severe pathological alterations characterized by diffuse lesions affecting more than 50% of hepatic lobules (score 3). In the prefrontal cortex, neuronal damage was evaluated based on the number of neurons showing degenerative changes characterized by pyknotic nuclei (Yeni et al., 2025). Mild injury (score 1) corresponded to 6–12 affected neurons, moderate injury (score 2) to 13–18 affected neurons, and severe injury (score 3) to 19 or more affected neurons observed in the examined fields. (Hassan et al., 2024).

### 2.7 Immunohistochemical examination

Immunohistochemical analyses were conducted according to the protocol described by previous literature (Karadogan et al., 2025). Briefly, paraffin-embedded prefrontal cortex brain tissue sections were deparaffinized, rehydrated, and subjected to antigen retrieval before incubation with the primary polyclonal antibody against glial fibrillary acidic protein (BT-Lab, Cat. No BT-AP03549; dilution 1/200) to specifically label astrocytic cells. Sections of both paraffin-embedded liver and brain tissue were deparaffinized and incubated overnight with primary antibodies (1/200 dilution) to Cleaved Caspase 3 (Thermo Fisher Scientific, Cat. No. PA5-114687) and Bax (Elabscience, Cat. No. E-AB-13814) to examine apoptotic activity. Secondarily, the large volume detection system: anti-Polyvalent HRP (Thermofischer, Catalog no: TP-125-HL) was applied as recommended by the manufacturer. 3.3′Diaminobenzidine (DAB) was used as the chromogen. After counterstaining with Mayer’s Hematoxylin, sections were covered with entellan and examined under a light microscope. Immunoreactivity was scored between 0 and 3.

### 2.8 Statistical analysis

The ‘SPSS 22.0’ (IBM, United States) program was used for statistical analyses. Quantitative data were shown as mean ± standard deviation. The Shapiro-Wilk test was used to confirm the normality of the biochemical data. Since the data was determined to be normally distributed, statistical analysis was performed using one-way ANOVA. The homogeneity of variance was assessed using the Levene test. Based on the test results, Tukey HSD and Games-Howell tests were preferred for *post hoc* tests. Since histopathological, immunohistochemical, and immunofluorescent data were ordinal, the Mann-Whitney U test was used for analysis. P < 0.05 was determined as statistically significant.

## 3 Results

### 3.1 TFA pretreatment attenuates TAA-induced hepatic injury

**FIGURE 1:**
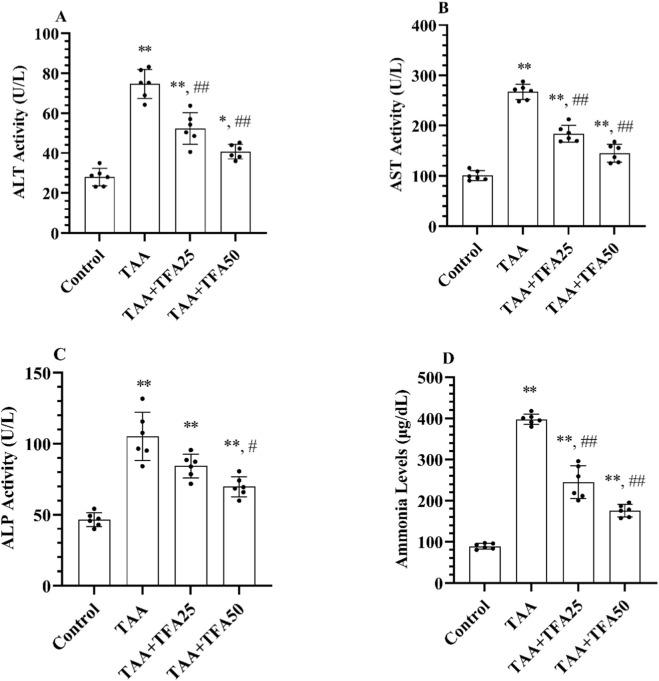
**(A-D)** Serum hepatic markers’ activities and ammonia concentration in control and treatment groups. Values are expressed as mean ± SD, n = 6 per group. **p < 0.001 vs. control group, *p < 0.01 vs. control group, ##p < 0.001 vs. TAA group, #p < 0.05 vs. TAA group. ALT: Alanine aminotransferase, AST: Aspartate aminotransferase, ALP: Alkaline Phosphatase, TAA: thioacetamide. TFA: Trans-ferulic acid.

As illustrated in Figures 1A–D, an increase in ALT, AST, ALP, and ammonia levels was detected in the serum samples of TAA-administered rats compared to the control, confirming hepatotoxicity (p < 0.001). Additionally, pre-treatment of TFA (at both 25 and 50 mg/kg doses) prevented the TAA-induced elevation in ALT, AST, ALP activities, and ammonia levels (p < 0.001). These results demonstrated that TAA induced hepatotoxicity in rats, while pre-treatment with TFA protected against it by reducing ALT (Figure 1A), AST (Figure 1B), ALP (Figure 1C) activities, and ammonia (Figure 1D) levels.

### 3.2 Histopathological evaluation of hepatic tissues

**FIGURE 2:**
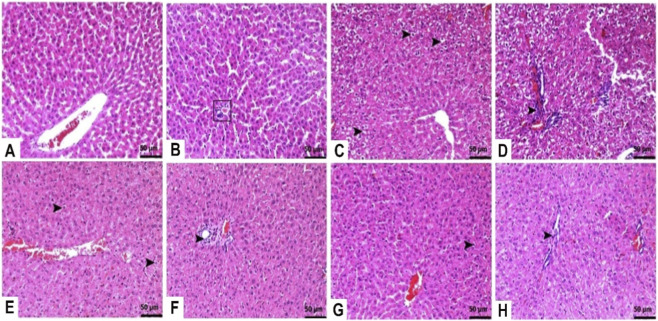
Effects of TFA on TAA-induced histopathological changes in liver tissues (Haematoxylin and eosin-stained images of the liver x 400). **(A)** Control: Normal histological appearance, **(B)** Control: Normal histological appearance of periportal areas (□), **(C)** TAA: Severe hydropic degeneration (►), **(D)** TAA: Severe mononuclear cell infiltrations in periportal areas (►), **(E)** TAA + TFA25: Moderate hydropic degeneration (►), **(F)** TAA + TFA25: Moderate mononuclear cell infiltrations in periportal areas (►), **(G)** TAA + TFA25: Mild hydropic degeneration (►), **(H)** TAA + TFA50: Mild mononuclear cell infiltrations in periportal areas (►). TAA: thioacetamide. TFA: Trans-ferulic acid.

Histopathological examination of liver tissues in control group samples revealed normal histological appearance (Figures 2A,B). However, TAA injection caused severe hydropic degeneration in the liver tissue and severe mononuclear cell infiltration in the periportal areas (Figures 2C,D). In contrast, both 25 mg/kg (Figures 2E,F) and 50 mg/kg (Figures 2G,H) TFA pre-treatment protected against TAA-induced liver damage (Table 1).

**TABLE 1:** Analysis results of histopathological data in the control and treatment groups.

Tissues	Hisropathological parameters	Animal groups (n = 6 per group)				P values
		Control	TAA	TAA + TFA25	TAA + TFA50	
		Mean ± standard deviation				
Liver	Hydropic degeneration	0	2.83 ± 0,40^a^	1.66 ± 0,51^b^	1.00 ± 0,00^c^	<0.05
	Mononuclear cell infiltration in the periportal area	0	2.66 ± 0,51^a^	1.66 ± 0,51^b^	0.83 ± 0,00^c^	<0.05
Brain	Degenerative changes characterized by pyknotic nuclei	0	2.83 ± 0,51^a^	2.00 ± 0,51^b^	1.00 ± 0,00^c^	<0.05

Histopathological grading; 0: none 1: mild damage, 2: moderate damage, 3: severe damage. Statistical analyses were performed using the Mann–Whitney U test. Within each row, groups marked with different lowercase letters (a–c) are significantly different from each other (p < 0.05). Groups that share the same letter do not show a statistically significant difference. TAA: thioacetamide. TFA: Trans-ferulic acid.

**FIGURE 3:**
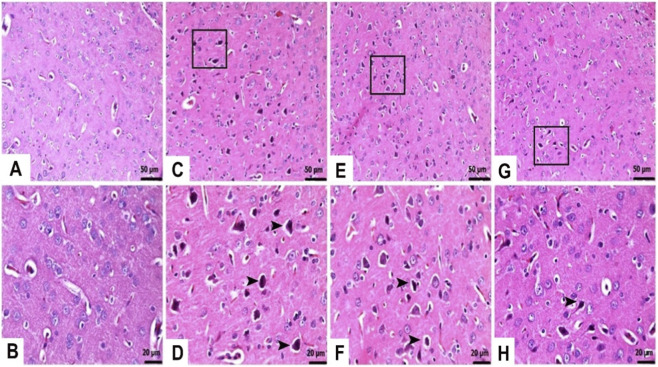
Effects of TFA on TAA-induced histopathological changes in brain tissues (Haematoxylin and eosin-stained images of the brain x 400). **(A,B)** Control: Normal histological appearance, **(C,D)** TAA: Severe degenerative changes characterized by pyknotic nuclei (□, ►), **(E,F)** TAA + TFA25: Moderate Degenerative changes characterized by pyknotic nuclei (□, ►), **(G,H)** TAA + TFA50: Mild degenerative changes characterized by pyknotic nuclei (□, ►). TAA: thioacetamide. TFA: Trans-ferulic acid.

As shown in Figures 3A,B, the brain tissue of the control rats exhibited normal histological appearance. In the TAA-administered group, severe degenerative changes characterized by were observed in the brain tissue samples (Figures 3C,D). However, both doses (25 mg/kg and 50 mg/kg) of TFA pre-treatment inhibited the TAA-related increase in neuronal degeneration in the brain tissue of rats (Figures 3E–H). Moreover, the 50 mg/kg TFA dose was more effective against the destructive effects of TAA (Table 1).

### 3.3 TFA pretreatment modulates TAA-induced serum proinflammatory markers

**FIGURE 4:**
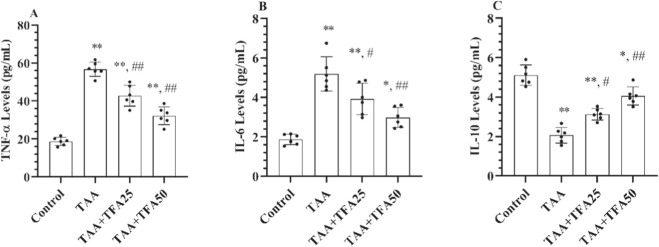
**(A-C)** Serum proinflammatory cytokine levels in control and treatment groups. Values are expressed as mean ± SD, n = 6 per group. **p < 0.001 vs. control group, *p < 0.05 vs. control group, ##p < 0.001 vs. TAA group, #p < 0.05 vs. TAA group. TNF-α: tumor necrosis factor-alpha, IL-6: interleukin-6, IL-10: interleukin-10, TAA: thioacetamide. TFA: Trans-ferulic acid.

In this study, serum TNF-α, IL-6, and IL-10 inflammatory markers were measured to reveal the possible effects of inflammation in rats with HE (Figures 4A–C). The results demonstrated increased serum concentrations of TNF-α and IL-6, while a reduced level of IL-10 was observed in the TAA-evoked rats according to control (p < 0.001), indicating a heightened inflammatory state. The pre-treatment with TFA (25 and 50 mg/kg) showed a marked decline in the levels of TNF-α and IL-6 concomitant with an elevation in IL-10 level, compared to the TAA alone group (p < 0.05).

### 3.4 TFA pretreatment suppresses TAA-induced astrocytic GFAP expression

**FIGURE 5:**
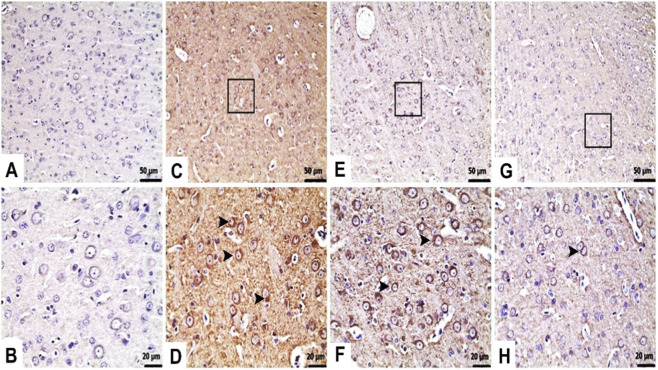
Effects of TFA on GFAP activation in the brain. Immunohistochemistry for GFAP. **(A,B)** Control: Immunonegative, **(C,D)** TAA: Severe, **(E,F)** TAA + TFA25: Moderate, **(G,H)** TAA + TFA50: Mild GFAP immunopositivity (□, ►). TAA: thioacetamide. TFA: Trans-ferulic acid.

GFAP, an astrocyte activation marker (França et al., 2019), was assessed in the brain tissue of rats from the experimental groups (Table 2). Immunoexpression of GFAP was at normal levels in control group brain tissues (Figures 5A,B), as opposed to this, exposure to TAA led to a marked elevation in the immunoexpression of this protein in the rat’s brain (p < 0.05, Figures 5C,D). Interestingly, pre-treatment of TFA (25 and 50 mg/kg) prevented elevated levels of GFAP with TAA, demonstrating its ameliorating features of astrogliosis (p < 0.05, Figures 5E–H). Moreover, this effect was most pronounced in the TAA group pretreated with 50 mg/kg TFA.

**TABLE 2:** Analysis results of immunohistochemical data in the control and treatment groups.

Tissues	Immunohistochemical parameters	Animal groups (n = 6 per group)				P values
		Control	TAA	TAA + TFA25	TAA + TFA50	
		Mean ± standard deviation				
Liver	Caspase 3	0	2.83 ± 0,51^a^	1.83 ± 0,40^b^	1.16 ± 0,40^c^	<0.05
	Bax	0	2.66 ± 0,51^a^	1.66 ± 0,51^b^	0.83 ± 0,40^c^	<0.05
Brain	GFAP	0.83 ± 0,40^a^	3.00 ± 0,00^b^	2.16 ± 0,40^c^	1.33 ± 0,51^d^	<0.05
	Caspase 3	0	2.83 ± 0,40^a^	2.16 ± 0,40^b^	1.33 ± 0,51^c^	<0.05
	Bax	0	2.66 ± 0,51^a^	1.66 ± 0,51^b^	0.66 ± 0,51^c^	<0.05

Immunohistochemical grading; 0: none 1: mild, 2: moderate, 3: severe, 4: very severe. Statistical analyses were performed using the Mann–Whitney U test. Within each row, groups marked with different lowercase letters (a–d) are significantly different from each other (p < 0.05). Groups that share the same letter do not show a statistically significant difference; TAA, thioacetamide; TFA, Trans-ferulic acid; GFAP, glial fibrillary acidic protein.

### 3.5 TFA pretreatment attenuates TAA-induced apoptosis

**FIGURE 6:**
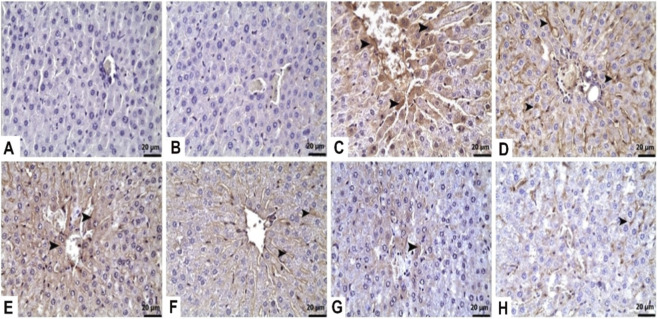
TFA represses TAA-induced apoptotic signaling activation in liver tissues. Immunohistochemistry for Caspase 3 and Bax. **(A)** Control: Immunonegative (Caspase 3), **(B)** Control: Immunonegative (Bax), **(C)** TAA: Severe Caspase 3 immunopositivity (►), **(D)** TAA: Severe Bax immunopositivity (►), **(E)** TAA + TFA25: Moderate Caspase 3 immunopositivity (►), **(F)** TAA + TFA25: Moderate Bax immunopositivity (►), **(G)**. TAA + TFA50: Mild Caspase 3 immunopositivity (►), **(H)** TAA + TFA50: Mild Bax immunopositivity (►). TAA: thioacetamide. TFA: Trans-ferulic acid.

**FIGURE 7:**
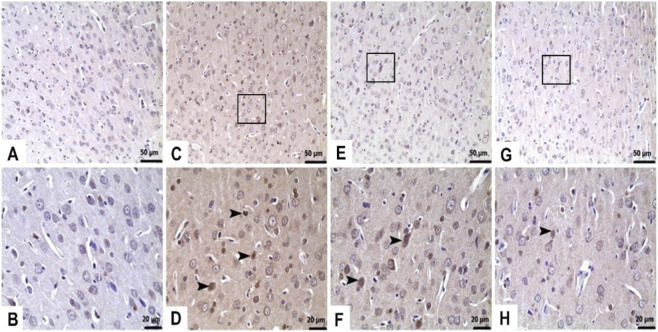
TFA represses TAA-induced apoptotic signaling activation in brain tissues. Immunohistochemistry for Caspase 3. **(A,B)** Control: Immunonegative, **(C,D)** TAA: Severe (►), **(E,F)** TAA + TFA25: Moderate (►), **(G,H)** TAA + TFA50: Mild Caspase 3 immunopositivity (►). TAA: thioacetamide. TFA: Trans-ferulic acid.

**FIGURE 8:**
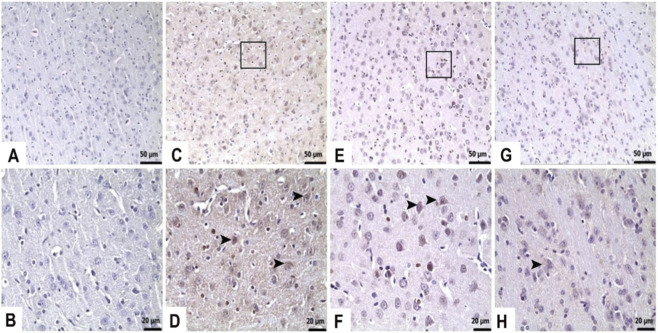
TFA represses TAA-induced apoptotic signaling activation in brain tissues. Immunohistochemistry for Bax, **(A,B)** Control: Immunonegative, **(C,D)** TAA: Severe Bax immunopositivity (►), **(E,F)** TAA + TFA25: Moderate Bax immunopositivity (►), **(G,H)** TAA + TFA50: Mild Bax immunopositivity (►). TAA: thioacetamide. TFA: Trans-ferulic acid.

Given the sustained activation of the MAPK/NF-κB pathway, which is known to promote inflammatory responses (Ali and Datusalia, 2024), and considering that chronic inflammation can contribute to the induction of apoptosis (Ali and Datusalia, 2024; Hassan et al., 2024), we subsequently assessed the expression levels of apoptotic markers, specifically Bax and Caspase-3, in liver and brain tissues (Figures 6–8; Table 2).

As shown in Figure 6 and Table 2, a significant increase in immunohistochemical staining of Caspase-3 and Bax was observed in the liver tissue of the TAA-only group according to the control (p < 0.05). To this end, TFA pre-treatment (25 and 50 mg/kg) prevented the apoptotic signalling viz., elevation in expression of Caspase-3 and Bax induced by TAA in the liver (p < 0.05).

As can be seen in Figures 7, 8 and Table 2, the immunohistochemical analysis also demonstrated a significant increase in Caspase-3 and Bax expression intensity in brain tissue of the TAA compared to the control (p < 0.05). Moreover, pretreatment with TFA at doses of 25 and 50 mg/kg effectively attenuated the expression of both apoptotic markers in the brain (p < 0.05).

### 3.6 TFA pretreatment inhibits TAA-induced inflammation via modulation of the MAPK/NF-κb signaling pathway

**FIGURE 9:**
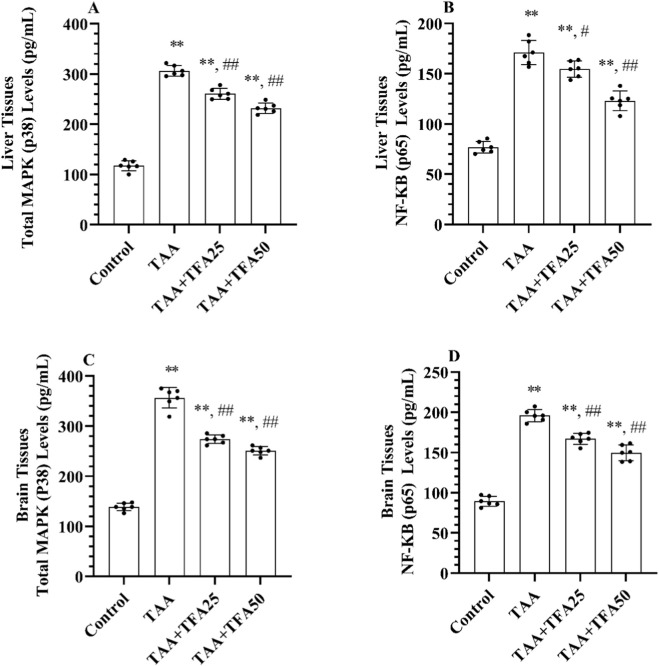
**(A-D)** Total MAPK and NF-κB (p65) levels in liver and brain tissues of control and treatment groups. Values are expressed as mean ± SD, n = 6 per group. *p < 0.001 vs. control group, #p < 0.001 vs. TAA group, ##p < 0.05 vs. TAA group. MAPK: mitogen-activated protein kinase, NF-κB: nuclear factor-kappa B, TAA: thioacetamide. TFA: Trans-ferulic acid.

To improve our understanding of the inflammatory signalling pathways involved with HE, we measured the levels of MAPK associated with the activation of the key inflammatory factor NF-κB (p65). The HE animals showed significantly higher levels of MAPK (p38) and pNF-κB (p65) in the liver (Figures 9A,B) and brain (Figures 9C,D) compared to the control group (p < 0.001)). Notably, pre-treatment with TFA (25 and 50 mg/kg) resulted in a dose-dependent reduction in the levels of NF-κB (p65) and MAPK in both liver and brain tissues compared to the TAA alone group (p < 0.05).

## 4 Discussion

HE is a severe neuropsychiatric disorder resulting from liver failure, significantly impairing prognosis and quality of life (Ali and Datusalia, 2025; Cordoba, 2014). Phenolic compounds have shown promise prophylactically in delaying HE onset and improving outcomes (Abd Elrazik and Abd El Salam, 2024; El-Hagrassi et al., 2022). The current study provides a new perspective on the efficacy of TFA in protecting against liver injury and neurological exacerbations associated with TAA-induced HE. Our results, for the first time, unveiled that TFA can alleviate TAA-promoted changes by reducing inflammation, glial cell activation, and apoptosis associated with modulation of the MAPK/NF-κB signaling pathway.

Current report, administration of TAA led to a marked elevation in serum aspartate AST, ALT, and ALP activities, reflecting hepatocellular leakage and disruption of hepatic membrane architecture. Moreover, HE development was confirmed by a significant increase in serum ammonia levels. These findings align with previous reports validating this model (Ali and Datusalia, 2024; Jia et al., 2020). Notably, TFA pre-treatment reduced these elevated liver enzymes and ammonia levels, confirming its hepatoprotective effect and enhancement of hepatic detoxification mechanisms (Mahmoud et al., 2020; Nouri et al., 2023).

Hyperammonemia is known to trigger pro-inflammatory cytokine production while suppressing anti-inflammatory responses, thereby contributing to the neurodegenerative processes characteristic of HE (Gallego-Durán et al., 2024; Ntuli and Shawcross, 2024). Moreover, circulating cytokines can infiltrate the impaired blood–brain barrier, initiating glial activation and amplifying production of a full repertoire of cytokines (Ali and Datusalia, 2025; Le Guennec et al., 2025). In the context of a potent inducer of inflammation (Ezhilarasan, 2023), application of TAA resulted in a balance shifted in favor of proinflammatory cytokines, as proven by markedly elevated levels of TNF-α, IL-6, and reduced IL-10 levels in serum. Our results align with prior experimental evidence indicating that TAA administration promotes inflammatory responses through the elevated of proinflammatory cytokine levels (Chen et al., 2008; Wang et al., 2011). In this study, TFA was chosen for its potent antioxidant and anti-inflammatory properties, forming the basis of our hypothesis that it may mitigate inflammatory processes in HE (Liu et al., 2025). Consistent with this, treatment with TFA exhibited potent anti-inflammatory effects, characterized by a significant increase in the anti-inflammatory cytokine IL-10 and reductions in TNF-α and IL-6 levels in serum. These results align with existing literature reporting marked anti-inflammatory activity of TFA in both *in vitro* and *in vivo* studies, further supporting its potential as a therapeutic agent in inflammatory conditions (Cao et al., 2015; Park et al., 2022).

Multiple signaling cascades, including MAPK and NF-κB pathways, are involved in modulating inflammatory responses in TAA-induced HE models (Ali and Datusalia, 2024; Huang et al., 2021). It was reported that upon activation, MAPKs and NF-κB translocate to the nucleus, orchestrating the transcriptional regulation of pro-inflammatory genes, which subsequently amplifies the neuroinflammatory cascade observed in TAA-related HE model (França et al., 2019). To develop a more thorough understanding of favorable effect of TFA on TAA-related liver and brain degeneration, we focused on the p38-MAPK/NF-κB signaling pathway, which is essential for regulating multiple cellular functions. Evidence from this study demonstrated that TAA drastically promoted elevation of p38-MAPK and NF-κB levels in the livers and brains of rats compared with those in the tissues of control experimental animals, in line with previously reported findings (Ali and Datusalia, 2024; França et al., 2019; Huang et al., 2021). Furthermore, we questioned whether TFA administration exerts a modulatory effect on MAPK/NF-κB-driven inflammatory pathways within the liver and brain of rats subjected to TAA-evoked HE. It is noteworthy that pre-treatment of TFA suppresses the substantial increase in the levels of p38-MAPK and NF-κB. These findings are consistent with previous reports demonstrating that TFA mitigates MAPK/NF-κB pathway activation in testicular tissue, thereby supporting the notion that its anti-inflammatory properties may be mediated through a common molecular mechanism across different organ systems (Hassanein et al., 2021).

Results from other studies have demonstrated that inhibition of MAPK and the use of antioxidants suppress NF-κB activation, alleviating neuroinflammation and ammonia-induced astrocyte edema (Bobermin et al., 2020; Sinke et al., 2008). Therefore, to further investigate astrocytic responses, we measured GFAP expression, a hallmark of astrocyte activation. Our findings revealed a significant upregulation of GFAP immunopositivity in the brain, indicating pronounced astrocytic activation. This increase was accompanied by molecular evidence of inflammation, mediated via the MAPK/NF-κB signaling pathway. Given the well-established role of these pathways in driving neuroinflammatory responses and associated histopathological alterations (Ali and Datusalia, 2024; 2025; França et al., 2019), our data suggest that MAPK/NF-κB activation may contribute to the observed astrocytic reactivity and tissue damage. Intriguingly, pre-treatment with TFA significantly decreased astrocyte activation alongside suppression of MAPK/NF-κB signaling. As anticipated, this reduction in neuroinflammation corresponded with protection against histological brain alterations, highlighting the anti-inflammatory properties of TFA (Ghobadi et al., 2022; Park et al., 2022).

Apoptosis in acute liver injury is closely associated with the inflammatory response. In TAA-induced models, pro-inflammatory cytokines and NF-κB activation have been shown to correlate with apoptotic markers, including caspase-3 and the Bax/Bcl-2 ratio (Chen et al., 2021). Moreover, in TAA-induced liver injury, early apoptosis is followed by inflammation-associated necrosis, and these 2 cell death pathways have been reported to proceed in a sequential and complementary manner (Ledda-Columbano et al., 1991). In this context, considering the well-established link between chronic inflammation and apoptotic cell death in neurodegenerative conditions (Rai and Bergmann, 2024), we aimed to investigate whether neuroinflammation in hepatic encephalopathy may trigger apoptotic signaling. Accordingly, Bax and caspase-3 were selected as key markers representing the intrinsic and execution phases of apoptosis, respectively, due to their established roles in mitochondrial dysfunction and cellular degradation (Tamnanloo et al., 2024). Our results revealed a clear upregulation of both Bax and active caspase-3, suggesting that sustained inflammatory signaling—likely mediated via MAPK/NF-κB pathways—may contribute to neuronal loss through apoptotic mechanisms. Consistent with our findings, prior research has indicated that upregulation of caspase-3 and BAX is a hallmark of apoptotic cell death in HE, particularly in rodent models (Ali and Datusalia, 2025; Okkay et al., 2022; Tamnanloo et al., 2024). Importantly, TFA pre-treatment not only attenuated astrocytic activation and inflammation but also reduced the expression of these apoptotic markers. This dual effect implies that TFA may exert protective properties by targeting both inflammatory and cell death pathways, potentially preserving tissue integrity in the context of HE.

The overall findings of the present study point to a coordinated regulatory effect of TFA on key molecular pathways implicated in the pathogenesis of hepatic encephalopathy. Rather than acting through a single target, TFA appears to influence interconnected signaling networks that govern inflammation and cell death. Given the well-established crosstalk between NF-κB and MAPK (p38) pathways, modulation of these signaling axes may disrupt the self-amplifying cycle of neuroinflammation and apoptotic damage. In this context, the simultaneous regulation of these pathways may help preserve cellular integrity in both hepatic and neural tissues. Notably, this experimental model closely reflects key pathophysiological features of hepatic encephalopathy observed in clinical settings, including hyperammonemia, neuroinflammation, and apoptosis. Therefore, the beneficial effects of TFA may be attributed to its capacity to fine-tune these complex molecular interactions, ultimately contributing to tissue protection. Collectively, these findings suggest that TFA may exert neuroprotective effects by targeting multiple pathogenic pathways, highlighting its potential as a promising translational candidate. However, further advanced preclinical validation and well-controlled clinical studies are warranted to confirm its clinical efficacy.

### 4.1 Limitations

A limitation of the present study is the absence of behavioral assessments, which could have yielded crucial functional correlations with the observed neuropathological changes. Although behavioral testing is instrumental in evaluating motor and cognitive impairments associated with neurodegeneration (França et al., 2019; Tamnanloo et al., 2024), in our laboratory, access to validated HE-specific behavioral platforms was limited, precluding inclusion of such assessments. Future research integrating both molecular and behavioral analyses would offer a more comprehensive understanding of disease progression and therapeutic efficacy. Another limitation of the present study is that microglial activation and proinflammatory cytokine levels in brain tissue were not evaluated. Inclusion of these analyses would have provided a more detailed understanding of the neuroinflammatory processes involved. Additionally, the absence of a group treated with TFA alone constitutes a limitation of the study.

## 5 Conclusion

In this study, the protective effect of TFA pretreatment was evaluated in a rat model of TAA-induced HE. TFA pretreatment effectively attenuated TAA-induced biochemical alterations, as evidenced by reductions in serum AST, ALT, ALP, and ammonia levels. This biochemical improvement was accompanied by a marked alleviation of TAA-associated hepatic histopathological damage. It also mitigated systemic inflammation by suppressing elevated TNF-α and IL-6 levels while preventing the decline in IL-10. Furthermore, TFA inhibited the activation of MAPK/NF-κB inflammatory signaling pathways in both liver and brain tissues and reduced GFAP expression in the brain. It also prevented the increase in pro-apoptotic markers, including caspase-3 and Bax, in both tissues. Overall, TFA emerges as a promising multi-target therapeutic strategy for alleviating hepatic dysfunction and neuroinflammation in HE.

## Data Availability

The original contributions presented in the study are included in the article/supplementary material, further inquiries can be directed to the corresponding author.
